# An Unexpected Anemia Hiding a Rare Syndrome With Overlapping Phenotypes

**DOI:** 10.14309/crj.0000000000000926

**Published:** 2022-11-24

**Authors:** Arianna Dal Buono, Laura Poliani, Alessandro Repici, Cesare Hassan, Paolo Bianchi

**Affiliations:** 1 Gastroenterology and Digestive Endoscopy Unit, Department of Gastroenterology, Humanitas Research Hospital, Rozzano (Milan), Italy; 2 Department of Biomedical Sciences, Humanitas University, Pieve Emanuele, Milan, Italy; 3 Gastroenterology and Endoscopy, IRCCS Ospedale San Raffaele, Milan, Italy; 4 Clinical Analysis Laboratory, Oncological Molecular Genetics Section, Humanitas Research Hospital—IRCCS, Rozzano (Milan), Italy

## Abstract

Gastric polyposis is a rare endoscopic finding that can imply genetic syndromes predisposing to cancer development. Among the possible conditions associated with gastric polyposis and early onset gastric cancer (younger than 45 years) is juvenile polyposis syndrome. We present a clinical case of early onset gastric cancer associated with a frameshift mutation in the gene *SMAD4*. Individuals carrying a pathogenic variant of this gene have a high risk of malignant transformation, especially of gastric cancer. Moreover, most of these patients present also with extraintestinal features of the hereditary hemorrhagic telangiectasia, and the first symptom prompting medical evaluation is anemia.

## INTRODUCTION

Gastric polyposis is a rare endoscopic finding that can be associated with genetic syndromes predisposing to cancer development in the gastrointestinal (GI) tract.^1,2^ Juvenile polyposis syndrome (JPS) is one of the possible hereditary disorders associated with gastric polyposis and early onset gastric cancer (younger than 45 years).^1,2^ JPS is a rare precancerous condition that presents with numerous juvenile polyps in the colorectum or in other GI locations. Concomitant extraintestinal manifestations, above all cutaneous manifestations such as telangiectasia, pigmented nevi, and skeletal stigmata, can be associated with JPS. Furthermore, clinical features of hereditary hemorrhagic telangiectasia (HHT) (ie, epistaxis, mucocutaneous telangiectasia, and liver/brain/pulmonary arteriovenous malformation [AVM]) can be observed in patients with JPS. We present an unusual clinical case of early onset gastric cancer associated with a frameshift mutation in the gene *SMAD4*.

## CASE REPORT

**Figure 1.**
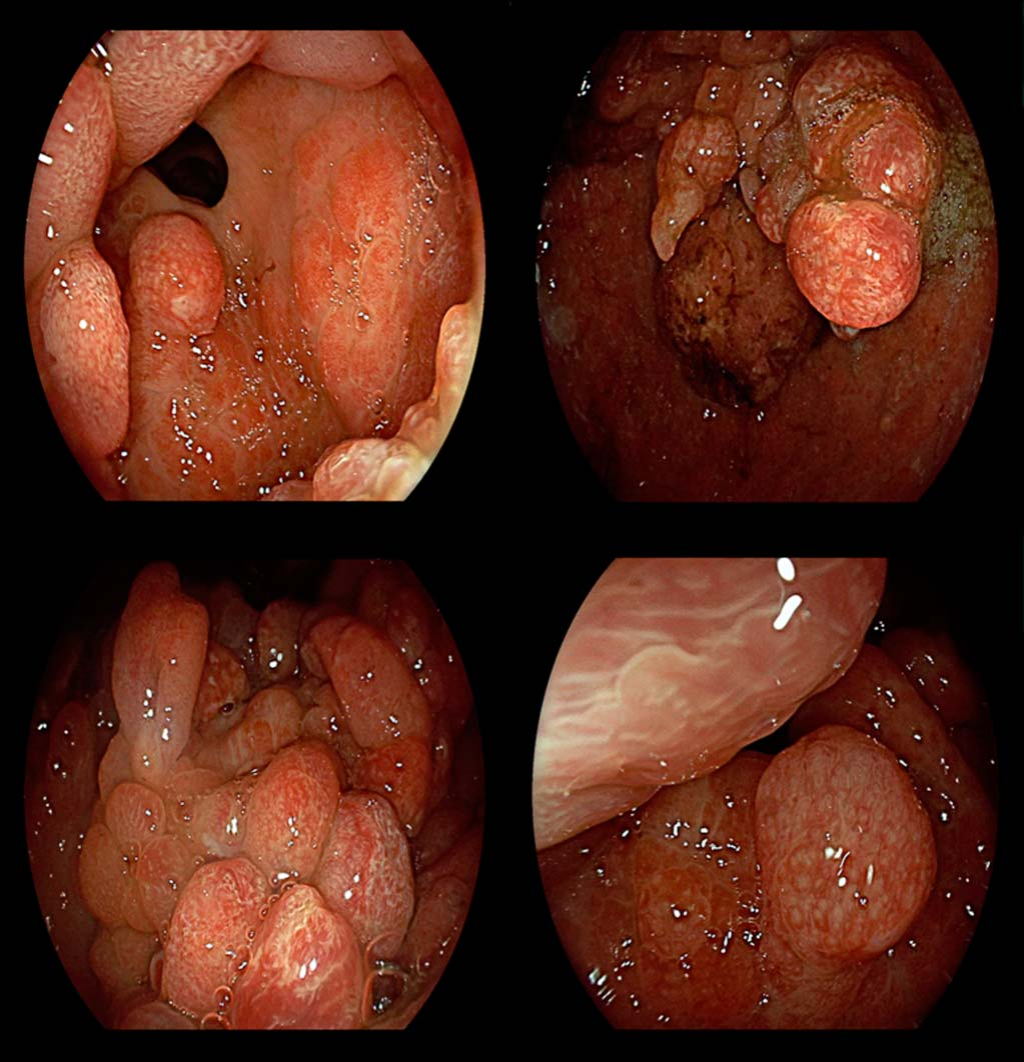
Endoscopic appearance of massive gastric polyposis associated with *SMAD4* mutation.

**Figure 2.**
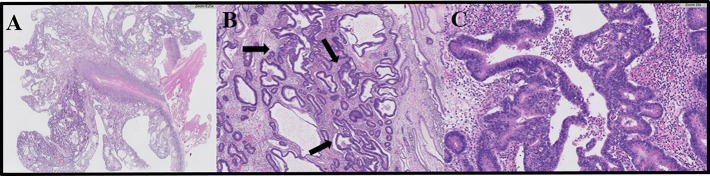
Microscopic features of intramucosal adenocarcinoma developing in the context of the gastric polyposis. (A) The histologic appearance of the intramucosal adenocarcinoma developing in the context of the gastric polyposis, H&E. (B) At higher magnification, neoplastic glands showing structural atypia (arrows) (H&E). (C) Detail of a neoplastic gland with complex architecture, including cribriform structures with necrosis, and cytologic atypia (H&E). H&E, hematoxylin and eosin stain.

A 45-year-old woman was referred for genetic counseling after a recent total gastrectomy with esophagojejunal anastomosis performed for a previous finding of high-grade dysplasia/intramucosal adenocarcinoma at multiple endoscopic biopsies of the stomach. The first upper endoscopy was performed a few months ahead in another hospital because of severe microcytic anemia (Hb values persistently less than 10 g/dL) without any further relevant symptoms (ie, melena and abdominal pain), where the whole gastric surface was described to be involved by numerous polyps, some ulcerated, that were biopsied and resulted in gastric adenomas with high-grade dysplasia/intramucosal adenocarcinoma. Thereafter, the patient underwent a computed tomography (CT) scan and endoscopic ultrasonography, which confirmed a diffuse gastric wall thickening with suspected areas of infiltration in the submucosa, without any detectable infiltration to the muscular layer. Before the scheduled surgical resection, an upper endoscopy was repeated at our institution showing the clinical picture of massive gastric polyposis (Figure 1). The histologic assessment of the surgical resection confirmed the presence of multiple gastric adenomas with foci of low-grade, high-grade dysplasia and multiple foci of intramucosal well-differentiated adenocarcinoma (pTis, N0, R0). Moreover, the histopathological examination of the polyps reported outer smooth surfaces with many dilated cystic glands, consistent with juvenile polyps (Figure 2). In addition, considering the finding of gastric polyposis, in the presurgical assessments, the patients had undergone a colonoscopy without evidence of colonic polyps or other causes of anemia. No evaluation of the small bowel was performed among the presurgical assessments because it was not relevant for the immediate oncological management.

Regarding her family history, her grandfather and one aunt from the maternal side died because of gastric cancer both in the sixth–seventh decade of age, whereas her father died at the age of 39 years because of colorectal cancer; she does not have siblings and has 2 sons of age 11 and 9 years.

Despite a significant family history of GI cancers, the patients had not undergone any endoscopic screening in the past.

Considering the age at onset of the gastric tumor (younger than 45 years), her family history with second- and third-degree affected members, and the underlying gastric polyposis, a genetic test was proposed to the patient. Target genes were comprehensively analyzed by next-generation sequencing (TruSight Cancer kit with TruSight Rapid Capture technology and the MiSeq platform, Illumina, San Diego, CA), and a germline frameshift mutation in *SMAD4* on exon 3, causative of a truncated or absent protein, was identified (NM_005359.6: c.293dup [p.Trp99LeufsTer5]) (see Figure 3, Supplementary Digital Content 1, http://links.lww.com/ACGCR/A30). In the post-test counseling, the genetic test and the appropriate endoscopic surveillance were proposed for all first-degree relatives, at the proper age (>15 years).

At her last follow-up, 6 months after the surgery, the patient had fully recovered, without any evidence of recurrence. The postsurgical follow-up in the next years will involve a yearly esophagogastroduodenoscopy and CT scan.

## DISCUSSION

We described a patient with massive gastric polyposis with an initial degeneration to adenocarcinoma associated with a germline mutation of *SMAD4*. The identification of this pathogenic variant determines a rare autosomal dominant disorder, the JPS. JPS has an estimated cumulative CRC risk that reaches 68%, and a cumulative risk of around 21% of upper GI tumors, including gastric cancer.^1,2^

A pathogenetic variant either in *BMPR1A* or *SMAD4* is detectable in around 40%–60% of the probands with suspicious clinical features.^3^ Both genes were recognized to encode proteins that are activated during the transforming growth factor-β signaling pathway, which regulates vital processes as proliferation, differentiation, and cellular death.^4,5^

As compared to those patients with JPS carrying a mutation of *BMPR1A*, *SMAD4* variants have been associated with a superior risk of gastric cancer, up to 30%, and with a generally more aggressive behavior of gastric polyps.^6,7^ Nearly 30% of probands with JPS have no informative or suspicious family history for polyposis, developing, therefore, a de novo mutation.^4^

Approximately 76% of these individuals can present some clinical features of the HHT, such as epistaxis, mucocutaneous telangiectasia, and liver/brain/pulmonary AVM.^8^

Several cases of concomitant JPS and HHT have been described in the literature; this association was formally ascertained by Gallione et al in their series of 7 families segregating both JPS and HHT phenotypes.^9–13^ Since then, further studies have provided data supporting that patients with *SMAD4* mutations exhibit this overlapped syndrome.^14–16^ JPS can be diagnosed through clinical criteria such as the presence of more than 5 juvenile polyps in the colon/rectum, and/or the presence of multiple juvenile polyps throughout the GI tract, and/or any number of juvenile polyps with a family history of juvenile polyposis and then confirmed by the genetic testing (Table 1). Clinical suspicion of HHT can be raised in case of recurrent and spontaneous epistaxis, multiple telangiectasias on the skin of hands, lips, face, nasal or oral mucosa, or visceral lesions (ie, AVMs of lungs, brain, liver, spinal cord, or GI) (Table 1).

**Table 1.** Clinical criteria for JPS and HHT

For JPS, one of the following must be present
More than 5 juvenile polyps in the colon/rectum
Multiple juvenile polyps throughout the GI tract
Any number of juvenile polyps with a family history of juvenile polyposis
The *Curaçao* criteria for HHT (definite HHT if 3 criteria are met, possible HHT with 2 criteria met)
Recurrent and spontaneous epistaxis
Multiple telangiectasis on the skin of hands, lips, face, or in nasal or oral mucosa
Visceral lesions (ie, AVMs of lungs, brain, liver, spinal cord, or GI)
A first-degree relative who meets the criteria

AVM, arteriovenous malformation; GI, gastrointestinal; HHT, hereditary hemorrhagic telangiectasia; JPS, juvenile polyposis syndrome.

Despite HHT was not initially suspected in our case, in the post-test counseling, considering the specific *SMAD4* mutation identified, we referred the patient for diagnostic work-out of HHT.

An additional association between *SMAD4* mutations and thoracic aortopathy has been reported, especially with aortic root dilation.^17,18^ Most pathogenetic variants of *SMAD4* are unique in every family, and to date, there is no evidence to support that specific variants of *SMAD4* might be more strongly associated with JPS-HHT. Hence, a wide range of variants including missense, nonsense mutations, and large deletions have been identified along the entire gene in these patients.^19^

Current international guidelines instruct on adequate surveillance recommending an upper endoscopy every 1–3 years and a colonoscopy to be performed annually.^20,21^

The endoscopic prevention program is advised from the age of 12–15 years.^20,21^ In addition, a small bowel exploration through capsule endoscopy and/or CT enterography can be considered, although the scarcity of the data is not mandatory in JPS.

This diagnosis of JPS should be suspected in any case of mixed, hypertrophic, and polypoid gastropathy; a comprehensive multigene panel (ie, *PTEN*, *STK11*, *APC*, *MUTYH*, MMR genes, *BMPR1A*, and *SMAD4*) should be offered in these cases. Of note, the endoscopic appearance of the gastric polyposis related to *SMAD4* mutations differs from the gastric adenocarcinoma and proximal polyposis of the stomach, where the antrum is usually not involved.

As concerns the prognosis of gastric polyposis within the different hereditary syndromes (familial adenomatous polyposis, MUTYH-associated polyposis, attenuated familial adenomatous polyposis, and JPS), the natural history and the true transformative rate are still unknown.

To conclude, proper genetic counseling and multidisciplinary management in tertiary referral centers are advisable for JPS patients with or without concomitant HHT and for their families.

## DISCLOSURES

Author contributions: A. Dal Buono performed the research and wrote the manuscript. L. Poliani, A. Repici, and C. Hassan critically reviewed the content of the paper. P. Bianchi conceived the subject of the paper, contributed to the critical interpretation, supervised the project, and is the article guarantor. All authors approved the final version of the manuscript.

Financial disclosure: None to report.

Informed consent was obtained for this case report.
